# Hannover study on long-stay hospitalization – part II: Characteristics and care conditions of long-stay hospitalization in cases of chronic mental illness

**DOI:** 10.1186/1745-0179-3-27

**Published:** 2007-11-26

**Authors:** Stefan M Bartusch, Bernd R Brüggemann, Hermann Elgeti, Marc Ziegenbein, Wielant Machleidt

**Affiliations:** 1 Department of Social Psychiatry and Psychotherapy, Hannover Medical School, Hannover, Germany

## Abstract

**Background:**

Long-stay hospitalization is often a consequence of insufficient care structures. This article examines the characteristics and care conditions of long-stay hospitalization (LSH) in an urban area in Germany.

**Methods:**

Extensive data of patients in the urban catchment area of the Medical School of Hannover, capital of Lower Saxony, were evaluated during a 10 years period.

**Results and conclusion:**

Community psychiatric efforts certainly help to reduce long-stay hospitalization, but cannot fully prevent it. Reference figures are given for comparable urbanized areas: consequently 500 chronically mentally ill persons per 100.000 inhabitants must be expected, 20% of which must be considered as long stay hospitalized according to a given definition. We estimate 250 places per 100.000 inhabitants to be required for institutionalised outpatient care, further 30 places for day clinic and full-time in-patient treatment and 40 places for residential home treatment. We suggest these results as a guidance for psychiatric planning in comparable communities.

## Background

Extensive reform processes based on the principles of the National Psychiatry-Enquiry of 1975 [1] and the Report Of The Expert Commission released in 1988 [2] have been initiated [3] since the middle of the 80s in Germany. These aims remain valid today. The implementation of the reform processes has, however, left some unsolved problems [4].

In the case of long-stay patients moved from psychiatric hospitals into big residential homes far away from the patients' community [5] specific planning figures are required [6] to supply the needs of all groups and to avoid inappropriate measures such as hospitalizations determined by the inavailability of resources or a place being only available outside the community.

Other European countries have acquired a much better experience with psychiatric community care systems than Germany. In England and Wales, for example, 33 acute in-patient beds are needed per 100.000 population [7] and in Italy only 17 [8]. This low figure in Italy is due to fundamental changes in the Italian psychiatric care systems under the 1978 reform legislation. In Germany the number of beds in psychiatric hospitals has decreased between 1970 and 1994 [9,10]. In the hanseatic city of Bremen, which has a similar number of inhabitants and a similar social structure in comparison to Hannover, Kruckenberg *et al*. [11] report a regional figure of 69 part-time and full-time in-patient treatment places per 100.000 inhabitants in 1992.

In nearly all federal German states the number of places in nursing institutions exceeds the number of places in therapeutic institutions. On principle it is legitimate to suggest a number of unreported cases of mentally ill persons living in non-psychiatric nursing homes for the elderly, women's shelters, shelter for the homeless and in prisons [12]. In spite of determined efforts, the city of Bremen still requires 20 places in temporary and permanent residental homes and 70 places in special nursing departments in hospitals [11]. Melchinger [13] reported a number of 46 places in psychiatric residental homes per 100.000 population existing within the area of Verden, Lower Saxony.

The number of in-patient treatment places in homes and hospitals is strongly dependent on the availability of outpatient and daily care capacities. Outpatient treatment institutions therefore need enough manpower to give intensive treatment for a defined catchment area and, if necessary, also in the patients' homes. In England and Wales the recommended figure for skilled employees working in psychiatric outpatient facilities per 100.000 inhabitants is 20,6 [14]; an inquiry among 27 (not necessarily comparable) outpatient institutions in Germany showed an average of only 4.12 skilled employees in 1994 [14].

Long-stay patients are more and more being recognized as a remaining problem for contemporary psychiatry [15-36] and also under economic aspects [37].

The outpatient, in-patient and complementary care services included in this study have been available to the chronically mentally ill living in the evaluated catchment area for more than 25 years. The conditions of treatment in these facilities remained stable during this period. This study examines the group of chronically mentally ill persons with general psychiatric diagnoses who are long-stay patients in hospitals, residential homes or nursing facilities and their conditions of treatment. We suggest a definition for long-stay hospitalization, and evaluate factors which indicate a complicated course or a disadvantageous outcome [38,39]. The evaluated care conditions and characteristics may serve as a basis for prevention of long-stay hospitalization.

## Methods

### The area and institutions

The examined area in this study is the psychiatric catchment area (Sector 5) of Hannover (capital of Lower-Saxony, Germany) served by the Department of Social Psychiatry and Psychotherapy of the Hannover Medical School since the 70s [40]. Hannover has approximately 500.000 inhabitants living in an area of 204 square kilometres. Sector 5 is the smallest area of all 10 sectors of the regional community psychiatric network, but is densely populated. From 1977 to 1997 the population decreased slightly to 63.000 inhabitants. A characterization of the social structure based upon the data of a census in 1987 [41] shows higher-than-average urban density and an above-average social situation.

Since the beginning of psychiatric reformatory efforts these institutions have played an important role in the development of outpatient and complementary services in Hannover. Since its opening in 1977 the Social Psychiatric Policlinic of Hannover Medical School integrates the tasks of an official community psychiatric service and of a medical treatment centre. Psychotherapeutic, sociotherapeutic, pharmacological and rehabilitational treatment methods are applied in close co-operation with general practitioners, specialists, social institutions and the authorities. The therapeutical concept and the working conditions remained unchanged over the years.

### The study group

The study group was established under the following criteria:

- contact to one of the institutions included in the study (see above) between 1987 and 1996,

- a period of at least two years between the first and the last contact to one of the three institutions mentioned (independent of a continuous use of the services),

- aged between 18 and 60 (at first contact),

- resident within the catchment area (Sector 5 of Hannover),

- a primary psychiatric diagnosis excluding addictions or psycho-organic disorders (e.g. dementia or mental handicap).

The archives of medical records of the Social Psychiatric Policlinic were the most important source of data, being the institution with which most of the patients (92%) had primary contact. The complete patients records of the other two institutions were also evaluated. In the case of equivocal data, the corresponding therapists were consulted or the medical records from relevant in-patient treatments were viewed.

The following anonymous characteristics were collected in a data base for each patient of the study group:

Permanent data: sex, date of birth, year of onset of psychiatric disease, year of first contact to one of the institutions mentioned, if differing: basic data of the time of first psychiatric-therapeutical contact, in cases where the treatment ended during the period of the study: year and reason of termination of treatment.

Temporary data: basic data of each year of contact (first diagnosis, duration since onset of disease, living situation, work situation, primary income, address of residence), data of service of each year of contact: method and duration of institutional psychiatric supports (number of quarters for outpatient and complementary supports, number of days for the time of partial and full-time in-patient treatment).

The temporary data were collected for each year between 1987 and 1996 in which at least one contact with the evaluated institutions had occurred. If the treatment was continued into 1997 the complete data of 1997 were registered. In cases with the first contact before the year 1987, the basic data of the year of first contact was also included in the data base.

Using the characteristic features of the psychiatric base data we composed a scaled sum score (psychosocial risk score = PSR; scale 1 to 4) for each patient and each year This score (PSR) evaluated at onset of treatment proved to be predictive of a disadvantageous course of the disease. As to the composition and calculation of the psychosocial risk score see Part I [38].

Common definitions or criteria for LSH refer to longstanding treatment in hospitals for one ore more years, but there is no established and consistent definition. We suggest an extended definition: in this study a person is considered to be long-stay hospitalized if he or she matched at least one of the following criteria during the period of the study:

- duration of more than 365 days of in-patient treatment within two successive years (independent of the number of in-patient stays),

- living in a psychiatric residental home for at least four quarters within two successive years,

- transfer to a long-stay in-patient nursing institution.

All the data recorded were entered into SPSS for Windows (version 12.0) and evaluated using the available statistical procedures such as descriptive analysis, non-parametric tests (Kruskal-Wallis test, Wilcoxon signed rank test, Mann-Whitney) and chi-squared test. Significant differences were found for p < 0.05, and high significance was assumed for p < 0.01.

## Results

### Total group

Data of 313 patients were included in the study. Further characteristics are found in table 1 (see also [38]). As to the involved institutions (policlinic, hospital, complementary institution) in 1991 the proportion of patients with a residence in the evaluated area at the time of their first therapeutical contact was 87% for the policlinic, 31% for the psychiatric hospital and 11% for the facilities of the complementary society.

**Table 1:** Total group vs. group with long-stay hospitalization (LSH)

	Total group	Group with LSH
First diagnosis at first therapeutical contact		
- ICD-10: F4	16%	6%
- ICD-10: F6	16%	12%
- ICD-10: F2, F30.1, F30.2, F31, F32.2, F32.3, F33.2, F33.3	68%	82%
Sex ratio *		
- female	62%	47%
- male	38%	53%
Age of onset of disease **		
- average	30 years	27 years
- < 25 years	38%	56%
- 25 – 44 years	51%	7%
- 45 – 59 years	11%	37%
Period between onset of disease and first therapeutical contact^n.s.^		
- average	6 years	5 years
- < 1 year	31%	25%
- 1 until < 5 years	26%	32%
- 5 until < 10 years	17%	13%
- >10 Jahre	26%	30%
Residence at first therapeutical contact **		
- in catchment area	83%	68%
- outside catchment area	17%	32%
Situation of living at first therapeutical contact^n.s.^		
- self-supporting, not alone	56%	52%
- self-supporting, alone	37%	29%
- in therapeutical institution	5%	16%
- homeless	2%	3%
Situation of employment at first therapeutical contact **		
- full-time job	47%	28%
- part-time employment	4%	0%
- in therapeutical institution	4%	7%
- unemployed	46%	65%
Primary income at first contact **		
- own income	34%	10%
- insurance payments	26%	30%
- relatives	27%	35%
- on social security	13%	25%
PSR at first therapeutical contact **		
- average	2,3	2,6
- low psychosocial risk	16%	6%
- moderate psychosocial risk	43%	31%
- significant psychosocial risk	38%	57%
- high psychosocial risk	3%	6%

* p < 0,05 ** p < 0,01 ^n.s. ^not significant (p > 0,05)

Analysis of the temporary data (table 2) showed the following trends: on comparison of the first with the second five-year period significant differences were found for age (patients grew older), the longer period since the onset of disease, the proportion of persons suffering from psychoses increased.

**Table 2:** Study group – results of variant data

Period (average)		1987–1991		1992–1996	
		**N**	***%***	**N**	***%***

**Number of patients**		184	*100*	191	*100*
**Sex**	Male	81	*44*	76	*40*
	Female	103	*56*	115	*60*
Age **	< 25 years	11	*6*	4	*2,2*
	25–44 years	96	*52*	97	*51*
	45–64 years	68	*37*	73	*38*
	> 64 years	9	*5*	17	*8,9*
First diagnosis * (ICD-10)	F4	18	*10*	13	*7*
	F6	24	*13*	24	*12*
	F2, F30.1, F30.2, F31, F32.2, F32.3, F33.2, F33.3	142	*77*	154	*80*
Period since onset of disease *	< 1 year	9	*5*	2	*1*
	1 – 5 years	32	*17*	24	*12*
	5 – 10 years	36	*20*	40	*21*
	>10 years	107	*58*	126	*66*
Outpatient care ** (quarters)	1	24	*13*	19	*10*
	2	18	*10*	11	*6*
	3	18	*10*	21	*11*
	4	79	*43*	98	*51*
quarters/patient		3,0		3,3	
patients/year		139	*76*	149	*78*
In-patient and day-clinic Care * (days)	< 10	8	*5*	4	*2*
	10 – 30	15	*17*	14	*17*
	31 – 91	31	*35*	33	*41*
	> 91	32	*36*	26	*33*
days/patient		86		86	
patients/year		89	*48*	80	*42*
**Residential home treatment** (quarters)	1	2	*7*	2	*9*
	2	4	*16*	1	*5*
	3	2,0	*8*	1,4	*7*
	4	17	*68*	15	*78*
quarters/patient		3,1		3,5	
patients/year		25	*13*	19	*10*
**Work Rehabilitation** (quarters) **	1	4	*17*	3	*12*
	2	3	*13*	2	*9*
	3	3	*14*	1	*5*
	4	12	*56*	19	*73*
quarters/patient		3,1		3,4	
patients/year		21	*11*	26	*14*

* p < 0,05 ** p < 0,01

The total number of patients increased slightly during the course of the study. In the policlinic they were treated more and more intensively (from 3.0 to 3.3 quarters/year). The number of in-patient (also day clinic) treatments in the psychiatric hospital decreased, the average duration of in-patient treatments showed no change (86 days/year).

The proportion of patients treated in a psychiatric residential home was reduced (from 25 to 19 patients/year), the average duration did not change. The number of those taking advantage of offers of work and occupational rehabilitation increased together with the continuity of utilization. The number of patients who were transferred to external residential homes for the elderly or to nursing homes increased progressively during the course of the study, reaching 21 patients in the year 1996.

### Classification according to duration of hospitalisation

We divided the study population into five groups according to the degree of hospitalization during the course of the study (table 3). The degree of hospitalization was defined by the sum of all treatment days in a psychiatric hospital in the evaluated period divided by the number of the years of treatment per patient during the study.

**Table 3:** Several characteristics of the group according to degree of hospitalisation

	group 1 (no hospitalization)	group 2 (<10 days/y.)	group 3 (10 – 30 days/y)	group 4 (>30 days/y)	LSH-group	total group
N =	63	56	60	66	68	313
coming from Sector 5	86%	88%	88%	86%	68%	83%
Female	67%	64%	58%	71%	47%	62%
PSR at first contact (score)	2,1	2,1	2,1	2,3	2,6	2,3
non-organic psychoses	50%	70%	72%	96%	93%	76%

We found differences between the five groups in the sex ratio, the number of non-organic psychoses, the psychosocial risk (PSR) at the time of the first therapeutical contact and the use of the outpatients' clinic.

The quota of patients living within the catchment area was steady at a high level in the groups without long-stay hospitalization. In the LSH-group significantly less patients originally came from the evaluated area. There are similar results as regards the sex ratio: In the groups without LSH the number of women dominates and in the LSH-group the proportion of men is slightly higher. The PSR in the year of first therapeutical contact increased with the degree of hospitalization; the highest value being consistently achieved in the LSH-group; an increase of the values is also found in the percentage of non-organic psychotic disorders.

### Group with long-stay hospitalisation

The group with LSH includes 22% (N = 68) of the study group. The LSH-group can be divided into three subgroups (table 4): the first subgroup consists of patients with long-stay in-patient treatment in hospital, subgroup 2 of patients hospitalized in psychiatric residential homes and subgroup 3 of patients living in conventional nursing homes or homes for the elderly.

**Table 4:** Subgroups of LSH-group

	Subgroup 1 (only hospital treatment)	Subgroup 2 (treated in therapeutic residential homes)	Subgroup 3 (treated in nursing homes and homes for the elderly)
N =	8	39	21
Female	38%	41%	67%
PSR at first contact (score)	2,6	2,7	2,7
coming from Sector 5	100%	28%	80%
non-organic psychoses	88%	90%	95%

Subgroup 1: eight patients (38% women) entered the LSH-group by the hospital criterion alone without having received care in a residental or nursing home or residential home for the elderly during the 10-years-period. At the time of their first therapeutical contact all these patients lived in the catchment area and did not differ from the total group of LSH as to their average PSR. This subgroup consisted mainly of recently diagnosed patients demanding a very intensive treatment. Nearly all (N = 7) patients were suffering from non-organic psychoses.

Subgroup 2: 39 patients (women 41%) had been treated in a therapeutic residential home for at least four quarters in two years without the necessity of a subsequent treatment in a nursing home. Only 28% of these patients lived in the catchment area at the time of their first therapeutical contact. 90% of the patients in this subgroup suffered from non-organic psychoses. The average PSR at the time of the first therapeutical contact (= 2.7) was clearly elevated.

Subgroup 3: During the 10-years-period 21 patients transferred into nursing homes and homes for the elderly. 67% were women, 80% of the patients lived in the catchment area at the time of the first therapeutical contact. The majority suffered from a non-organic psychosis (95%). The PSR of this subgroup was as high as in subgroup 2 (= 2.7). The number of patients in nursing homes or homes for the elderly increased progressively during the course of the study. One patient was transferred due to physical illness, five patients were older than 60 years at the time of transfer. Three of the remaining 16 patients younger than 60 years did not live in the catchment area at the first therapeutical contact. The average age of the remaining 12 patients was 47 years in the year of transfer.

## Discussion and conclusion

The complete registration of patients' data in a developed care system for a 10-year-period is, to our knowledge, unique in Germany. The results give information about LSH-patients and suggest reference figures for the planning and evaluation of psychiatric care in community psychiatric networks in comparable regions. However, in some aspects even a 10-year-course evaluation delivers only limited results, for example, most of the recorded patients (76%) fell ill prior to the start of the evaluation period.

A total of 313 patients came into contact with one of the institutions involved during the 10-year-period of examination. 68 of them (22%) met the criteria for LSH. This group can be characterized as predominantly male, suffering mainly from non-organic psychoses and, at its first therapeutical contact, already combined with an elevated PSR [38]. A further distinction within this group is the difference in sex-ratio. The proportion of women is elevated with 67% for patients in closed or nursing homes. Patients living in psychiatric residential homes represent the majority of LSH-patients and mostly came originally from outside the catchment area. This is not the case with the most important group relative to LSH-prevention efforts characterized as subgroup 3: patients transferred into closed or nursing homes. Although serious somatic diseases may also have led to this placement, there are patients in this group who could no longer be treated in the community due to severe behavioural disturbances rendering them a danger to themselves or others. It is striking that the proportion of women is quite high, even slightly higher than in the total group. This agrees with similar results to those found in the literature [32], although it seems difficult to interpret.

The analysis of our temporary data shows a clear trend to more intensive and continuous outpatient treatment, and the simultaneous reduction of inpatient and home treatment capacities. The investigation of the institutions proved suitable therefore for the derivation of planning estimates. In calculating the demand for institutional psychiatric care two important points had to be considered. Firstly, some patients, younger then 60 years from the catchment area transferred into external nursing homes or residential homes for the elderly prior to the start of the study period. This group could not be taken into account. Consideration of these patients is, however, important for the estimation of the demand for psychiatric care. Secondly, patients treated for the first time during the last two years of the study could not be included in the study due to the defined exclusion criteria. Furthermore, we had no access to information concerning imprisonment of patients or their admission to forensic hospitals.

According to the results evaluated, based upon the study's criteria, 500 persons from 100.000 inhabitants must be expected to suffer from chronic mental illness (excluding primary addictions and psycho-organic disorders), who will consequently be using institutional community psychiatric care services. 280 of these patients are currently treated per year in these institutions. They utilize around 250 treatment places in the outpatients' clinic and 30 part- or full-time in-patient places in a hospital.

According to our empirical results a mobile community psychiatric outpatient service has to care for 225 chronically mentally ill adult patients (without addiction disorders, organic disorders) per 100.000 inhabitants three to four quarters a year, an additional 75 places for patients needing only temporary treatment are also necessary.

In addition to the above-mentioned 30 places for in-patient treatment of chronically mentally ill patients another estimated 20 hospital places for other patients including geriatric patients and addicts are to be expected on empirical grounds. To meet these requirements 0.5 in-patient beds per 1.000 inhabitants for patients older than 18 years are needed. This figure approximates to the data for England and Wales, but is below the German results (1.2 for 1997 [42]; approx. 0.7 for 2001 [43])

Under the circumstances described above, approximately 35 places in residential homes with individually adjusted intensity of treatment are necessary per 100.000 inhabitants for chronically mentally ill patients under the age of 60. This figure seems comparatively low for urban areas [9]. As long as there are no regional standards for community psychiatric services more people will move into regions with better conditions and the utilization of these services will rise. The avoidance of treatment in nursing or residential homes for the elderly entails flexible individual adjustment of therapeutic care to ensure a rehabilitative residential home treatment for people younger than 60.

Twelve patients younger than 60 years and living in the catchment area at first contact had to be transferred at an early date to a nursing or residential home for the elderly (average age = 47 years) because of an intensive need for – mostly somatic – care. This means a demand for 23 home places per 100.000 inhabitants. These 23 places are equivalent to 60% of the aforementioned regional demand of home places. The development of new forms of outpatient care could however lead to a lessening of the demand for care in residential homes.

The study data do not enable an estimate of the regional demand for places of specialized work rehabilitation and sheltered jobs since the availability of such offers was still insufficient within the examined catchment area. The study group required, on the basis of 100.000 inhabitants, an average of 27 places preferably in a sheltered workshop. Of these 27 places however, around 50% were occupied by patients who had moved into the catchment area initially to live in a therapeutic residential home.

A continuous outpatient treatment within a defined area with short distances between therapist and patient is decisive in the prevention of LSH. A corresponding mobile outpatient service should not, however, be specialized solely for the treatment of chronically mentally ill people. An extended responsibility for emergency cases should include the possible use of coercive measures to enable better access to risk patients. Furthermore a wide and differentiated spectrum of responsibilities might very well help to avoid the danger of „chronification" to the employees in this occupation.

In our experience some 14 full-time appointments (per 100.000 inhabitants) for qualified personnel including administrative staff are necessary to run an interprofessional emergency and regular outpatient service within the working hours. One therapeutic full-time employee has to care for about 24 patients per quarter besides tasks such as leading therapeutical groups, being on standby for emergencies and visiting team conferences for example.

It is certainly to be expected that LSH can be reduced by means of communal psychiatric measures but will not of course be fully prevented [20]. In a well-developed communal psychiatric care system around 20% of the patients stay long-term either in hospitals, therapeutic residential homes or nursing homes. The comparatively high rate of 20% for LSH-patients must be seen relatively to the extended LSH-criteria employed. Nevertheless we suggest our definition of LSH to be suitable and qualified for further scientific adoption.

The continuous treatment of patients with a high risk of LSH would seem possible in a sufficiently staffed outpatient after-care institution. A decrease of the capacities in partial and full-time in-patient psychiatric treatment as well as in residential homes was achieved by intensive effort.

## Competing interests

The author(s) declare that they have no competing interests.

## Authors' contributions

SMB and HE conceived and designed the evaluation, collected the clinical data, and helped to draft the manuscript. HE re-evaluated the clinical data and revised the manuscript. BRB performed the review of the literature. BRB and SMB performed the statistical analysis and revised the manuscript. MZ and WM re-analyzed the clinical and statistical data and revised the manuscript. All authohrs read and approved the final manuscript.
